# Impact of the COVID-19 Pandemic on the Work Engagement of UK Active Employees

**DOI:** 10.3390/healthcare10071226

**Published:** 2022-06-30

**Authors:** Macarena Romero-Martín, Juan Gómez-Salgado, Miriam Alcaide-Carrasco, Lucas Rodríguez-Jiménez, Mónica Ortega-Moreno, Daniel López-López, Carlos Ruiz-Frutos

**Affiliations:** 1 Department of Nursing, Faculty of Nursing, University of Huelva, 21071 Huelva, Spain; macarena.romero@denf.uhu.es; 2 Department of Sociology, Social Work and Public Health, Faculty of Labour Sciences, University of Huelva, 21071 Huelva, Spain; frutos@uhu.es; 3 Safety and Health Postgraduate Programme, Universidad Espíritu Santo, Guayaquil 092301, Ecuador; 4 Yeovil District Hospital NHS Foundation Trust, Yeovil BA21 4AT, UK; mirianosqui@hotmail.com; 5 Advanced Clinical Practitioner, Royal Free London NHS Foundation Trust, London NW3 2QG, UK; lucas.rodriguezjimenez@nhs.net; 6 Department of Economy, Faculty of Labour Sciences, University of Huelva, 21071 Huelva, Spain; ortegamo@uhu.es; 7 Research Health and Podiatry Group, Department of Health Sciences, Faculty of Nursing and Podiatry, Industrial Campus of Ferrol, Universidade da Coruña, 15403 Ferrol, Spain; daniel.lopez.lopez@udc.es

**Keywords:** work engagement, work environment, COVID-19, pandemic

## Abstract

The objective of this investigation was to describe the work engagement perceived by UK workers during the COVID-19 pandemic. A descriptive cross-sectional study was conducted. The sample included 1085 participants, aged 18 years and older, living in the UK during the COVID-19 pandemic, who were active workers. Data were collected using an online questionnaire and the UWES-9. They were analysed using descriptive statistics, a *t*-test for equality of means or ANOVA, and the Chi-squared Automatic Interaction Detection method. The mean value in the UWES-9 was 3.46 (SD = 1.11). Participants with lower satisfaction (21.8%) gave significantly low or very low UWES-9 scores in 58.5% of the cases. Greater work engagement was obtained with more resources and less conflict, risk, and stress. In cases where there had been contact with COVID-19, this was associated with slightly lower levels of work engagement. These results could motivate and guide companies to adopt risk prevention measures and protocols to return to normal working conditions after the initial crisis phase of the pandemic.

## 1. Introduction

The coronavirus pandemic 2019 (COVID-19) has posed a major threat to public health worldwide. According to the latest data collected by the World Health Organization, COVID-19 has been responsible for more than 180 million confirmed cases and approximately 4 million deaths worldwide [1]. In the case of the UK, 4.7 million cases of COVID-19 and 128,000 deaths have been reported. Numerous governments, including the UK, have been forced to impose strict measures, including lockdown for the population with the closure of schools and non-essential workplaces, maintenance of social distance, and/or travel restrictions to reduce the spread of the virus [2]. The report published by WHO in 2020 states that such measures could have psychosocial and mental health consequences for the population. The occupational risk and economic impact of COVID-19, as well as domestic violence, drug or alcohol use, and the media are considered to be mental health risk factors. In addition, the high rates of infection and mortality coupled with the lack of treatment, limited resources, excessive workload, and the difficulty in curbing transmission is causing high levels of fear and anxiety among the general population, as well as among health professionals [3].

The occupational consequences of the pandemic have differed across sectors. The health sector has experienced an increased intensity and a tightening of working conditions, while many other workers were forced to stop working, telework, or even lost their jobs [4]. The measures adopted to contain the pandemic involved an abrupt and imposed change in work practices with strong consequences for workers’ well-being and performance. Measures such as working from home, the use of virtual environments, or remote leadership have been accelerated, and, although already in place, these have been implemented without taking into account workers’ preferences, increasing their vulnerability [5].

Companies have made an effort to provide safe workplace environments by implementing symptom surveillance measures; outbreak investigation and response, including contact tracing and isolation of those infected; personal protective equipment; environmental adjustments such as improving ventilation, adding physical barriers to prevent physical contact, or environmental cleaning; educational and signage initiatives; and organisational changes such as facility zoning, entry restrictions, changes in assignments for high-risk workers, or facility closures [6]; however, despite workplace measures to respond to the COVID-19 crisis, it has been described how employees express a high level of fear and concern about the disease [7].

In this scenario of uncertainty due to the dramatic measures needed by the extraordinary circumstances of the pandemic, Work Engagement (WE) is a concept that could help to combat the vulnerable situation of workers. WE is a positive cognitive and emotional perception of the work environment that encompasses three dimensions: dedication (emotional), vigour (physical), and absorption (cognitive) [8]. Dedication refers to work integration, enthusiasm, inspiration, pride, a feeling of importance, and challenge. Vigour is characterised by dedication and effort at work which are related to high levels of energy and mental stamina. Absorption is defined as the ability to concentrate and immerse oneself in work [9]; thus, WE relates to the worker’s capacity for engagement and connectedness to meet work demands, creating this way a feeling of job satisfaction.

The factors that facilitate WE can be divided into three: organisational, work, and personal factors. Organisational factors are those related to the work environment, such as leadership, organisational structures, and social support [10]. On the other hand, the level of autonomy, availability of resources, job opportunities, feedback, or well-defined job role are related to work factors. Finally, there are personal factors such as personality or efficacy. Therefore, other factors, such as workload, extra time worked, or conflicting decision making can have a negative effect on WE [11].

Previous studies have described how WE increases workers’ job satisfaction, career satisfaction, job performance, job effectiveness, and wellbeing. In addition, it reduces burnout and job turnover intention [10,12]. Workers with higher WE tend to experience more team spirit, team efficacy, emotional stability, and less physical, cognitive, and emotional workload and job demands [13]. WE has also been associated with fewer work family conflicts, with less perceived job stress [14], and with greater resilience and empathy [15]. For all these reasons, WE can be considered a valuable resource for workers to cope with the unstable scenario generated by the health crisis.

This study was conducted during the COVID pandemic, which was an unprecedented health crisis for which governments were unprepared. The results could help describe the impact of this type of situation in the work environment. Specifically, this study aims to describe the influence of the pandemic on WE, and therefore, on the emotional well-being of workers. Identifying how the pandemic influences workers would help to adopt preventive measures for future unforeseen situations.

The present study aims to describe WE as perceived by the population during the COVID-19 pandemic and to identify its relationship with participants’ sociodemographic characteristics, work environment, and contact with the disease in England. In this article the authors will describe the methods followed, then we will present the obtained results, we will discuss these results with the available evidence, and finally, we will narrate our conclusions supported by the results.

## 2. Materials and Methods

### 2.1. Design

Observational, descriptive, cross-sectional study.

### 2.2. Participants

The present study involved 1085 people, recruited by a convenience sampling method, who agreed to participate on a voluntary basis. Inclusion criteria for the study were persons aged 18 years and older, resident in the UK during the COVID-19 pandemic, and in a situation of employment. Therefore, people who were not residing in the UK at the time of their participation in the study or who were not in active employment, e.g., students, unemployed, retired, etc., were excluded.

### 2.3. Study Variables and Measurement Instruments

The variables included in the study were socio-demographic variables such as sex, age, marital status, last completed studies, type of housing, employment, children, confinement, and self-perception of health. In addition, other variables such as work environment, contact with COVID-19, and Work Engagement of the participants were considered.

For the assessment of the socio-demographic variables, work environment, and contact with COVID-19, a self-developed questionnaire was designed. In relation to the work environment, 13 multiple choice questions were included in which the participants expressed their degree of agreement with a statement related to their work environment on a Likert-type scale from 1 to 10, where a value of 1 corresponded to strongly disagree and a value of 10 to strongly agree. In relation to contact history with COVID-19, 5 questions on the relationship with infected persons were included. The initial draft of the questionnaire was piloted on 20 participants who met the inclusion criteria for the study; these participants were selected for convenience, and were asked to rate the questionnaire in terms of clarity and comprehension. Participants were asked to complete the survey from different electronic devices. None of the participants expressed comprehension problems or doubts about what was being asked. There were also no reported faults in relation to the platform or design in the different devices used (personal computer, Tablet or Smartphone) by the participants; thus, no adjustments were required according to their feedback.

The short version of the Utrecht Work Engagement Scale (UWES-9) [16] was used to assess WE. This self-administered instrument consists of nine items with Likert-type response scales ranging from 1 (Never) to 7 (Always) and distributed along three dimensions: Vigour, which refers to the presence of high levels of energy and resilience, willingness to devote effort, not getting tired easily, and being persistent in the face of difficulties; Dedication, referring to the meaning or significance of the work, feeling enthusiastic, proud, and inspired by the work completed; and absorption, which refers to feeling happy and immersed in the work so that time goes by quickly and making the person forget what is going on around them. As an outcome measure, the score for each dimension was calculated by adding up the items in each dimension and dividing the result by the number of items in each dimension.

### 2.4. Procedure

Data collection was carried out online using the Qualtrics^®^ (Provo, UT, USA) survey platform. Participants completed the questionnaire from any electronic device with internet access. The answers were recorded anonymously on this platform in a web space that could only be accessed by researchers with a password.

The link to the questionnaire was distributed in two ways. On the one hand, once approval had been obtained from the Health Research Authority, the National Health Service Trusts were contacted and asked to collaborate in the distribution. In response, they agreed to send an invitation to participate to health professional group mailing lists, or included an announcement in the newsletter. Alternatively, the invitation to participate with the link was distributed via social media groups. In both cases, participants were encouraged to spread the questionnaire among their colleagues and friends to trigger a snowball effect. Data collection took place between December 2019 and April 2020.

### 2.5. Data Analysis

Descriptive measures were performed for variables collecting information on socio-demographic data, performance in the face of mobility restrictions, health perception, dimensions of the UWES-9 test, work environment and contact history. The *t*-test for equality of means or ANOVA, with Welch’s correction if the hypothesis of homoscedasticity was not fulfilled, which allowed us to determine the existence of significant differences in the three dimensions and in the total score of the UWES-9 test. To determine significant differences between groups, post-hoc tests were performed, specifically the Scheffe and Games–Howell tests, depending on the homogeneity or not of the variance. In the case of quantitative variables, the variables were categorised according to quartiles, and in all cases, the effect size was determined.

Finally, the Chi-squared Automatic Interaction Detection (CHAID) method was used to build a classification tree to detect which characteristics of the participants played a relevant role in WE. To do this, using the χ^2^ test of independence, predictors with the lowest adjusted *p*-value were searched, provided that this value was less than or equal to the pre-set significance level of 0.05. Analyses were carried out using SPSS 26.0 statistical software (IBM, Armonk, NY, USA).

### 2.6. Ethical Considerations

Participants read the Participant Information Sheet beforehand, which described the purpose of the study, its procedure, and also explained that participation in the study did not involve any risk or benefit to the participants; they were asked to confirm their willingness to voluntarily participate in the study through informed consent. In addition, they were informed of the possibility to withdraw from the survey at any time they wished to do so. Data were collected and recorded anonymously, maintaining the confidentiality of the information at all times. This study has the National Health Service (NHS) Health Research Authority approval, Integrated Research Application System (IRAS) project ID 283849, REC reference 20/HRA/3997.

## 3. Results

### 3.1. Socio-Demographic Data of the Sample

As can be seen in Table 1, the 1085 sample had a mean age of 44.8 years (SD = 11.7), 86.1% were female, 70.5% lived with a partner, 61.7% had university or higher-level studies, 81.6% claimed to have a house with an outside view (house with a balcony, terrace, yard, or garden), and 58.2% had children under 16 years of age. Regarding the type of occupation, 51.1% were public employees, 41.2% were workers in private companies, and 7.7% were self-employed. Finally, 63.2% perceived their health as good or very good in the last two weeks and 55.2% had been in confinement, although going out for work or shopping.

### 3.2. Descriptive Results of Work Engagement

Table 2 shows different descriptive measures of the data, establishing five categories based on quantiles (5, 25, 75, and 95) for the different dimensions and for the total score of the UWES-9 test. The dimension with the lowest mean values was vigour (2.87, SD = 1.28), followed by absorption (3.70, SD 1.13); the highest-rated dimension was dedication (3.81, SD = 1.26). Regarding the total values of the UWES-9 test, the mean value was 3.46 (SD = 1.11), with 50% of the central data being between the values 2.89 and 4.22, 5% of the data being less than 1.37, and another 5% resulting in greater than or equal to 5.30.

### 3.3. Relationship between Work Engagement and Socio-Demographic Variables

When contrasting the scores of the different dimensions and the total score of the UWES-9 test with the socio-demographic characteristics, significantly higher values were observed in people who were married or living with a partner, with children under 16 years of age, and with a very good perception of health in the last 14 days. The self-employed group also stood out, with significant differences being detected between public employees and workers in private companies, except in the absorption dimension. In terms of sex, men reported significantly higher vigour levels. The level of education, type of housing, and whether or not they were in confinement did not show significant differences in any of the dimensions, nor in the total score (Table 1).

### 3.4. Relationship between Work Engagement and the Work Environment

As shown in Table 3, in relation to the work environment, the highest scores correspond to the variables related to psychological support, both for healthcare staff and for family members or the general population; there are no significant differences between the categories. Found with a mean score of 8.1 (SD = 2.1 and SD = 2.3, respectively) are the variables related to the resources provided by the company for more efficient and safer work, with significant differences being detected in the total score of the UWES-9 test of each group; these differences are also significant between quartiles when referring to increased stress at work (mean = 6.8, SD = 3.0), the degree of satisfaction with the COVID-19 situation (mean = 6.4, SD = 2.5). Safety distance (mean = 6.4, SD = 2.5), and increased conflict caused by the situation (mean = 4.1, SD = 2.9), with the latter having the lowest scores, they differ significantly in quartiles three and two, respectively. In relation to the distance, there are no significant differences between quartiles two and three, but there are between them and the rest. Finally, with regard to risk, there are no significant differences between the first and fourth quartiles, nor between the first, second and third quartiles; however, there are significant differences between the fourth with the first and second.

### 3.5. Relationship between Work Engagement and Contact History

With regard to the contact history of the participants, Table 4 shows that, in most cases, no contact of more than 15 min had taken place at a distance of less than 2 m (61.5%), no direct contact (58.1%), no contact with a person or material suspicious of being infected (50.8%), and no contact with an infected family member (65.3%); however, 68.4% reported having or probably having had a partner infected with COVID-19. No differences in WE were detected when a family member was infected. In case a co-worker or a close contact were infected, the WE decreased significantly. In the case of contact of more than 15 min within 2 m, there were significant differences in the total score, which was repeated in the case of contact with a person or material suspicious of being infected and when the infected person was a co-worker.

### 3.6. Classification and Regression Tree for UWES-9

**Figure 1:**
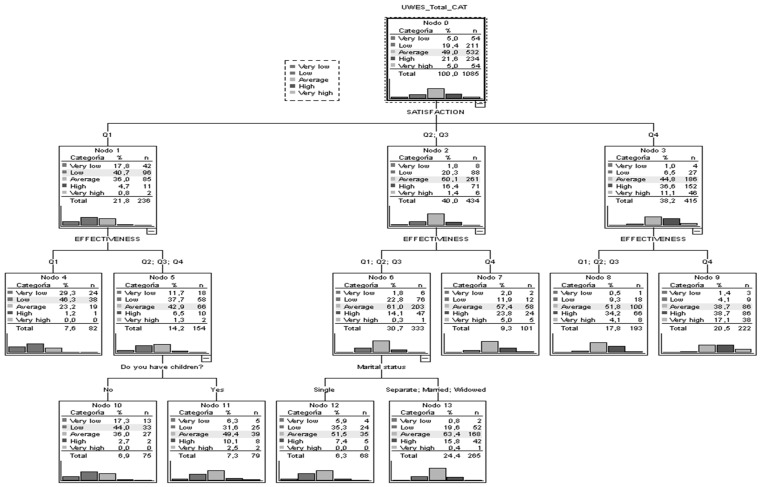
Classification and regression tree for UWES-9.

Job satisfaction is presented as the most significant variable with respect to the UWES-9 test score. Participants with lower satisfaction give low or very low test scores in 58.5% of the cases, while among those with higher satisfaction, give a high or very high total score (47.7%). Low satisfaction and feeling that one does not have sufficient resources for effective work (7.6% of cases) translates into 75.6% low or very low test scores. For those with low satisfaction and more resources for an effective job, the score is higher, but conditioned by whether or not they have children. If they do not have children (6.9%), low scores stand out, while those who have children (7.3%) score with mean values. For those participants with intermediate satisfaction (40%), the test score is mediated, as in the previous case, by resources for effective work and when these are not high by marital status; however, in all cases, the category with the highest proportion of cases is the mean, with a slight asymmetry to the left in the case of fewer resources for an effective job and being single. Finally, higher satisfaction and more resources lead to 55.8% of the participants scoring high or very high in the test, 38.7% intermediate, and the remaining 5.5%, low or very low (Figure 1).

## 4. Discussion

The aim of the present study was to describe WE felt by the UK population during the COVID-19 pandemic, and to identify associated factors related to the work environment and contact history of the workers. Participants reported a moderate level of WE, with the dedication dimension being the most highly valued with vigour being the least valued one. The WE identified was significantly higher in people with a partner, children under 16, self-employed, and with a very high self-perception of their own health. In relation to the work environment, psychological support and the resources offered to perform the job effectively and safely were particularly appreciated. The work environment has influenced WE during the pandemic. Workers reported significantly higher levels of WE the more resources they received, the more satisfied they felt with their work, the more they kept a safe distance, and the less conflict, risk, and stress they perceived. In terms of contact history, in most cases, no contact with COVID-19 was detected at the personal level, except among co-workers. Where contact did occur, it was significantly associated with slightly lower levels of WE.

The level of WE obtained in the present study (3.46, SD = 1.11) was similar to that obtained in other studies conducted during the pandemic, both internationally, 3.42 (SD = 1.12) [17], 3.56 (SD = 1.04) [18], and in the UK 3.88 (SD = 1.38) [19], although lower than that obtained in studies conducted in the UK prior to the pandemic 4.80 (SD = 0.93) [20]; these are inferred differences, as statistical differences were not explored, and this difference could be explained by the psychological impact of working conditions as a result of the pandemic. Job insecurity, adverse work environment, long periods of quarantine and isolation, exploitation of labour rights, and uncertainty of the future have worsened the mental health of workers, especially among young and highly educated workers [4]. Organisational climate, including leadership style and structural empowerment, and job resources, such as support, work environment, rewards, or satisfaction with organisational policies have been identified as influencing factors in WE [12]; these factors have been diminished during the pandemic, which may have triggered a decline in WE.

The dimension most highly rated by participants in this study was dedication (M = 3.81, SD = 1.26), as in previous studies [8,13,21]. Dedication refers to work integration, inspiration, and a feeling of importance. In this line, workers in the NHS have expressed a high level of commitment and dedication to work, feeling enthusiasm, excitement, and pride in the work they do despite the harshness of the health crisis [19]; their professional integrity has also been described with a cooperative and helpful attitude towards the institution, manifested in high levels of autonomy, optimism, and organisational citizenship behaviours, factors associated with higher WE [22]. As other authors pointed out, the high WE and job satisfaction reported by healthcare professionals reflects their conviction of the relevance of the work they perform and the importance of their contribution to the fight against COVID-19 [9].

Participants in our study recognised the need to offer psychological support to the public, those affected, and health professionals. These results are consistent with previous studies in which psychological support has been positively valued by workers during the pandemic, especially interventions such as cognitive behavioural therapy, motivational interviewing, and crisis intervention [4]. It was suggested that initiating psychological care from primary care with a comprehensive psychosocial assessment that includes COVID-19-related stressors (such as exposures to infected sources, infected family members, bereavement, and physical detachment), secondary adversities (economic loss), psychosocial effects (such as depression, anxiety, psychosomatic concerns, insomnia, increased substance use, and domestic violence), and indicators of vulnerability (such as pre-existing physical or psychological problems) [23] could be beneficial. Another investigation proposes early intervention through psychological crisis intervention and psychological first aid to manage the crisis emergency and buffer distress during the outbreak [24]; these strategies can help emotionally distressed survivors through practical help, contact, involvement, safety and comfort, and in addressing stress-related reactions. In addition, interventions aimed at strengthening resilience to cope with the psycho-emotional challenges of the pandemic have been proposed, including exposure to the outdoors, exercise, maintaining contact and support from family, friends, and significant others, better sleep, and more frequent prayer [25]. Appropriate use of technologies, the internet, and specifically social media as a means of reliable information, and the design of structured websites and toll-free helpline numbers have also been suggested to alleviate psychological distress among the general public regarding the pandemic [26]. In relation to WE, a study assessed the effect of staff wellness centres set up in a hospital setting to address the psychological impact of COVID-19 on health care workers [19]. These facilities were comfortable, quiet spaces with an upbeat atmosphere, which provided the opportunity to rest, relax, and unwind from the pressures derived from coronavirus. The results of the study revealed that workers who made use of these wellness centres reported significantly higher levels of WE.

The results of this study suggest that company-provided resources generate more WE in workers. These results are in line with those found by Sasaki et al. who identified that measures taken by companies to cope with the pandemic were associated with less psychological distress and higher employee work performance, thus protecting employees [7]. There is evidence to support that work-related stress aggravates mental health problems; however, measures to prevent contagion in work environments, the promotion of safe protocols, and the availability of personal protective equipment appear to moderate mental health risk and promote better performance and well-being of workers [4]. Workers demand a work environment that conveys confidence and minimises the risk of contagion. Another study on return to work after the pandemic found a low prevalence of anxiety, depression, stress, and insomnia among workers [27]; moreover, the adoption of infection prevention measures such as hand hygiene, face mask use, and reinforcement of workplace hygiene measures was associated with milder psychiatric symptomatology. In other words, workers are committed to their work performance, and their concern is focused on a safe, disinfected, and prevention-conscious workplace.

According to the results of our study, workers who perceived conflict and stress at work reported lower levels of WE. The relationship between job stress and WE is consistent with the one found in previous studies [10,12,28]. Workers with less WE have been identified as to be experiencing stress perceived at work in all its manifestations: harassment, overload, irritability, tension, fatigue, fear, anxiety, and lower energy, self-fulfilment, and satisfaction [28]. A study on intensive care professionals identified a negative relationship between cognitive and emotional demands and WE [13]. One of the main causes of stress at work is work overload, which has been described as a barrier to WE. When work demands exceed the resources available to workers, they perceive the work activity as an obstacle and a burden that undermines their WE [29]. In contrast, a stress-free work environment, including sufficient resources, good peer relations, effective management and leadership, and active worker participation promotes WE [21]. Mindfulness-based interventions in highly stressful work environments have been reported to increase resilience and WE [30]. The study by Zheng et al. revealed that the stressful effects of COVID-19 have negative effects on WE that can be buffered by mindfulness practice [18]. WE has a mediating effect between stress and burnout; the more stress is perceived, the lower the WE, leading to higher levels of emotional exhaustion and depersonalisation due to lack of engagement and loss of mental energy and stamina [31]. During the pandemic, a negative correlation has been identified between burnout and WE; the more burnout workers suffer, the lower WE they experience [32].

In relation to conflict, there is evidence demonstrating its relationship with WE. The study by Mache et al. revealed that work family conflict negatively correlates with WE [14]. The pressure of not being able to balance work and family roles satisfactorily reduces work engagement and job satisfaction. Interpersonal relationships at work have an effect on WE. Another study identified that a non-coercive power style, which is based on the attractiveness of exemplary job performance by the person of reference, increases WE [33]. It has also been identified that the more emotional intelligence people have, especially in interpersonal aspects, the more committed they feel to work, with more energy and mental resilience [34].

Our results indicate that exposure to risk and contact with illness depletes participants’ WE. These findings contradict the study by Blake et al. on the well-being of hospital workers during the COVID-19 pandemic, where workers in areas of higher risk and exposure to the virus reported higher WE than those with less exposure [19]; however, in a study in the United States with non-healthcare workers, the least committed to work were those who were most exposed to health risks [35]. According to the results found by other authors, the fear of workers exposed to COVID-19 reduces their job satisfaction and increases their psychological distress [36]. The availability of safe procedures to manage the risk of infection and the availability of personal protective equipment seems to moderate the risk of developing mental health problems [4].

In the present study, job satisfaction was found to be the most significant variable with respect to WE, which is consistent with previous studies [37,38]. Job satisfaction is understood as the feeling of satisfaction and pleasure provided by work, which can positively affect people’s lives [37]. Therefore, it is to be expected that people who enjoy their work activity with pleasure are more committed to their work; furthermore, this relationship could be explained by the job demands-resources model. According to this model, all jobs include both demands and resources, and trigger two independent psychological processes. On the one hand, a stress process, as job demands can lead to burnout, with a consequent deterioration of health. On the other hand, a motivational process driven by personal and work resources generates WE and job satisfaction [39,40]. Two authors identified that the relationship between WE and job satisfaction is moderated by role stress, i.e., when role ambiguity and role conflict are high, the influence of WE on job satisfaction is diminished [38]. Lack of clarity about job tasks, lack of role information, inadequate job descriptions, and incompatible role demands in the company negatively affect job performance and, consequently, committed employees feel less satisfied at work. In general, employees are more engaged when they work in an environment that provides practical and psychologically meaningful support, as well as opportunities for advancement and promotion [37].

As limitations to the present study, the non-randomised sample should be acknowledged, so caution is advised in generalising the results. In relation to sex, the sample was not homogeneous, and the online distribution of the questionnaires resulted in an uneven territorial distribution of the sample, which may have conditioned the results. An information bias must also be acknowledged due to the use of a self-completed questionnaire in the data collection.

## 5. Conclusions

According to the findings, a moderate level of WE was detected during the pandemic, with lower levels of energy and mental stamina reported by participants. Higher levels of WE were associated with providing resources to work safely and effectively, keeping a safe distance, and job satisfaction. On the other hand, WE was reduced when perceiving conflict, risk, stress, or being in contact with potentially infected people or material.

The results of this study help to describe the impact of the COVID-19 pandemic on people’s working lives. In the light of this study, workers are committed to their job performance, although their concern is focused on their workplace; these results could motivate and guide companies to adopt risk prevention measures and protocols to return to normal working conditions in case similar situations occurred. The provision of resources and the creation of safe work environments that include psychological support to help reduce work-related stress and potential conflict are suggested here; these measures would encourage WE with consequent benefits for both the worker and the company.

The pandemic has led to a transformation of workplaces that has affected people’s mental health. A return to a positive attitude, enthusiasm, and commitment to work is necessary for the psychological well-being of workers. In spite of the pandemic, workers maintain a predisposition to continue with work activity that provides them with satisfaction, well-being, and a sense of usefulness.

## Figures and Tables

**Table 1:** UWES-9 dimensions related to sociodemographic variables (N = 1085).

		Vigour			Dedication			Absorption			UWES-9		
	N (%)	M (SD)	Statistical	Effect Size	M (SD)	Statistical	Effect Size	M (SD)	Statistical	Effect Size	M (SD)	Statistical	Effect Size
Sex													
Female	934 (86.1)	2.8 (1.3)	−2.197 *	0.20	3.8 (1.3)	−1.373	0.12	3.7 (1.1)	−0.810	0.07	3.4 (1.1)	−1.630	0.15
Male	151 (13.9)	3.1 (1.4)			4.0(1.3)			3.8 (1.2)			3.6 (1.2)		
Marital status													
Single	201 (18.5)	2.5 (1.3)	8.649 **	0.02	3.5 (1.4)	5.893 **	0.02	3.4 (1.1)	6.950 **	0.02	3.1 (1.1)	9.065 **	0.03
Married or living with a partner	765 (70.5)	3.0 (1.3)			3.9 (1.2)			3.8 (1.1)			3.6 (1.1)		
Separate or Divorced	104 (9.6)	2.8 (1.3)			3.7 (1.3)			3.5 (1.2)			3.3 (1.2)		
Widowed	15 (1.4)	2.9 (1.3)			3.9 (0.9)			3.6 (1.0)			3.5 (0.8)		
Level of studies													
No studies	25 (2.3)	3.1 (1.5)	1.065	0.01	3.9 (1.6)	1.432	0.01	3.5 (1.5)	1.514	0.01	3.5 (1.5)	1.278	0.01
Secondary school	80 (7.4)	2.9 (1.1)			3.7 (1.2)			3.7 (1.1)			3.4 (1.0)		
High school	53 (4.9)	2.7 (1.4)			3.4 (1.5)			3.3 (1.4)			3.1 (1.3)		
Professional training	257 (23.7)	2.9 (1.3)			3.8 (1.3)			3.7 (1.2)			3.5 (1.2)		
University studies (undergraduate or degree)	431 (39.7)	2.9 (1.3)			3.8 (1.2)			3.7 (1.1)			3.4 (1.1)		
University studies (Master or Doctorate)	239 (22.0)	3.0 (1.3)			4.0 (1.2)			3.8 (1.0)			3.6 (1.1)		
Housing													
Apartment with balcony/terrace/patio	66 (6.1)	3.0 (1.1)	2.018	0.01	3.8 (1.2)	0.653	0.00	3.6 (1.0)	0.760	0.00	3.5 (1.0)	1.040	0.00
Apartment without balcony/terrace/patio	86 (7.9)	2.6 (1.2)			3.6 (1.3)			3.6 (1.2)			3.3 (1.1)		
House with garden/patio	885 (81.6)	2.9 (1.3)			3.8 (1.3)			3.7 (1.1)			3.5 (1.1)		
House without a garden/patio	29 (2.7)	2.5 (1.3)			3.7 (1.1)			3.5 (1.1)			3.2 (1.0)		
Other	19 (1.7)	2.7 (1.0)			4.0 (0.9)			3.9 (1.0)			3.5 (0.8)		
Employment													
Self-employed	84 (7.7)	3.5 (1.4)	14.440 **	0.03	4.3 (1.3)	7.447 **	0.01	4.0 (1.2)	2.856	0.01	3.9 (1.2)	9.742 **	0.02
Civil servant	554 (51.1)	2.7 (1.3)			3.7 (1.2)			3.7 (1.1)			3.4 (1.1)		
Private company worker	447 (41.2)	3.0 (1.2)			3.8 (1.3)			3.7 (1.1)			3.5 (1.1)		
Children < 16													
Yes	631 (58.2)	3.0 (1.2)	4.821 **	0.30	3.9 (1.2)	3.540 **	0.22	3.8 (1.1)	3.469 **	0.21	3.6 (1.1)	4.350 **	0.27
No	454 (41.8)	2.7 (1.3)			3.7 (1.3)			3.6 (1.1)			3.3 (1.1)		
Are you carrying the confinement decreed by the Government?													
Yes, in strict confinement	62 (5.7)	3.1 (1.5)	1.358	0.00	4.1 (1.4)	1.206	0.00	4.0 (1.2)	2.140	0.00	3.8 (1.3)	1.789	0.00
Yes, going out to buy and/or work	599 (55.2)	2.8 (1.2)			3.8 (1.2)			3.7 (1.1)			3.4 (1.1)		
I am not in any confinement	386 (35.6)	2.9 (1.3)			3.8 (1.3)			3.7 (1.1)			3.5 (1.1)		
Other situations	38 (3.5)	3.0 (1.1)			3.9 (1.2)			3.8 (1.1)			3.5 (1.1)		
Self-perception of health in the last two weeks													
Very poor	12 (1.1)	1.4 (1.9)	37.249 **	0.12	2.9 (1.9)	15.966 **	0.06	3.4 (1.4)	5.071 **	0.02	2.6 (1.6)	19.061 **	0.07
Poor	72 (6.6)	2.1 (1.3)			3.3 (1.2)			3.5 (1.2)			3.0 (1.1)		
Average	316 (29.1)	2.3 (1.2)			3.5 (1.3)			3.5 (1.2)			3.2 (1.1)		
Good	427 (39.4)	2.9 (1.2)			3.9 (1.2)			3.7 (1.1)			3.5 (1.0)		
Very good	258 (23.8)	3.5 (1.2)			4.2 (1.2)			3.9 (1.1)			3.9 (1.1)		

* *p* < 0.05; ** *p* < 0.001; M = median, SD = Standard Deviation.

**Table 2:** Dimensions and total score UWES-9 (N = 1085).

		Vigour	Dedication	Absorption	Total UWES
Range		0–6	0–6	0–6	0–6
Median		3	4	3.67	3.44
Mean		2.87	3.81	3.70	3.46
SD		1.28	1.26	1.13	1.11
Very low	<P_5_	<0.67	<1.33	<1.67	<1.37
Low	[P_5_, P_25_)	[0.67, 2)	[1.33, 3)	[1.67, 3)	[1.37, 2.89)
Average	[P_25_, P_75_)	[2, 3.67)	[3, 4.67)	[3, 4.33)	[2.89, 4.22)
High	[P_75_, P_95_)	[3.67, 5)	[4.67, 6)	[4.33, 5.67)	[4.22, 5.30)
Very high	≥P_95_	≥5	≥6	≥5.67	≥5.30

SD = Standard Deviation.

**Table 3:** UWES-9 score related to the work environment (N = 1085).

	Work Environment		UWES-9					
	P_25_/P_50_/P_75_	Mean (SD)	Q1	Q2	Q3	Q4	Statistical	Effect Size
Effectiveness	7/9/10	8.1 (2.1)	2.9 (1.2)	3.3 (0.9)	3.5 (0.9)	3.9 (1.1)	41.089 **	0.12
Safety	7/9/10	8.1 (2.3)	3.0 (1.2)	3.3 (0.9)	3.5 (0.9)	3.8 (1.2)	31.152 **	0.09
Distance (n = 671)	5/7/8	6.4 (2.5)	3.0 (1.2)	3.4 (1.1)	3.3 (1.0)	3.8 (1.1)	18.913 **	0.08
Contact (n = 414)	5/8/10	7.1 (3.2)	3.4 (1.1)	3.4 (1.0)	3.7 (1.0)	3.4 (1.2)	3.355 *	0.01
Conflict	1/3/6	4.1 (2.9)	-	3.7 (1.1)	3.3 (1.0)	3.3 (1.1)	17.723 **	0.03
Risk	2/7/9	6.0 (3.4)	3.7 (1.1)	3.6 (1.0)	3.4 (1.0)	3.3 (1.2)	5.628 **	0.02
Acceptance	3/6/8	5.8 (3.2)	3.5 (1.1)	3.6 (1.1)	3.4 (1.0)	3.4 (1.2)	2.041	0.01
Psycol. Support 1	8/10/10	9.0 (1.8)	3.5 (1.1)	3.4 (1.0)	3.5 (1.1)	-	1.462	0.00
Psycol. Support 2	8/10/10	8.9 (1.7)	3.6 (1.1)	3.4 (1.0)	3.4 (1.2)	-	1.870	0.00
Psycol. Support 3	8/10/10	8.6 (2.0)	3.6 (1.1)	3.4 (1.0)	3.5 (1.2)	-	2.041	0.00
Burden	5/8/10	7.0 (3.1)	3.6 (1.1)	3.5 (1.1)	3.5 (1.0)	3.3 (1.2)	2.306	0.01
Stress	5/8/10	6.8 (3.0)	4.0 (1.1)	3.7 (1.0)	3.4 (0.9)	3.0 (1.2)	40.939 **	0.11
Satisfation	5/7/8	6.4 (2.5)	2.5 (1.1)	3.3 (0.9)	3.5 (0.7)	4.1 (0.9)	122.711 **	0.29

* *p* < 0.05; ** *p* < 0.001. Rank of work environment variables 1–10. The variables related to the work environment have been categorized by quartiles, distinguishing the intervals [minimum, P_25_), [P_25_, P_50_), [P_50_, P_75_) y [P_75_, maximum]. **Effectiveness.** Do you think that your department, service, unit or company has provided you with the resources (material and means) necessary to carry out your work effectively? **Safety.** Do you think that your department, service, unit or company has provided you with the resources (materials and means) necessary to carry out your work safely? **Distance.** Do you think the distancing with your peers is adequate in preventing the transmission of coronavirus? **Contact.** Are you in contact with clients/patients that could be at risk of coronavirus transmission? **Conflict.** Have you noticed an increase in conflict at work from self-isolating? **Risk.** Do you feel that your job or workplace puts you at great risk of exposure to COVID-19? **Acceptance.** To what extent does your role put you at risk of catching COVID-19 infection? **Psycol. Support 1.** Do you think it would be important if there was a service offering psychological support to healthcare professionals and voluntary staff who are directly dealing with the health crisis of COVID-19? **Psycol. Support 2.** Do you think it would be important if there was a service offering psychological support to people affected by COVID-19 and their relatives, on facing difficulties caused by the health crisis? **Psycol. Support 3.** Do you think it would be important if there was a service offering psychological support to general population, on facing difficulties caused by the health crisis? **Burden.** Have you noticed an increase in your workload since the health crisis? **Stress.** Do you feel more stressed at work? **Satisfaction.** How would you rate the degree of satisfaction with your work in the current situation of COVID-19?

**Table 4:** UWES-9 dimensions related to contact record (N = 1085).

		Vigour			Dedication			Absorption			UWES-9		
	N (%)	M (SD)	S	ES	M (SD)	S	ES	M (SD)	S	ES	M (SD)	S	ES
Contact > 15′ < 2 m with infected person													
Yes	305 (28.1)	2.7 (1.2)	3.318 *	0.01	3.7 (1.3)	2.453	0.01	3.6 (1.1)	2.811	0.01	3.3 (1.1)	3.445 *	0.01
No	667 (61.5)	2.9 (1.3)			3.9 (1.2)			3.7 (1.1)			3.5 (1.1)		
Does not know	113 (10.4)	3.0 (1.4)			3.9 (1.4)			3.8 (1.3)			3.6 (1.2)		
Close contact with an infected person													
Yes	338 (31.2)	2.7 (1.3)	4.069 *	0.01	3.7 (1.3)	4.288 *	0.01	3.6 (1.1)	3.103 *	0.01	3.3 (1.1)	4.662 *	0.01
No	630 (58.1)	2.9 (1.3)			3.9 (1.2)			3.7 (1.1)			3.5 (1.1)		
Does not know	117 (10.8)	3.0 (1.4)			3.9 (1.2)			3.8 (1.1)			3.6 (1.1)		
Any contact with a person or material suspected of being infected													
Yes	364 (33.5)	2.7 (1.3)	3.967 *	0.01	3.7 (1.3)	2.875	0.01	3.6 (1.1)	1.250	0.00	3.3 (1.1)	3.187 *	0.01
No	551 (50.8)	3.0 (1.3)			3.9 (1.2)			3.7 (1.1)			3.5 (1.1)		
Does not know	170 (15.7)	2.9 (1.3)			3.9 (1.3)			3.7 (1.2)			3.5 (1.2)		
Any COVID-19 infected relative													
Yes	345 (31.8)	2.8 (1.3)	0.737	0.00	3.8 (1.3)	0.165	0.00	3.7 (1.2)	0.210	0.00	3.4 (1.2)	0.306	0.00
No	708 (65.3)	2.9 (1.3)			3.8 (1.2)			3.7 (1.1)			3.5 (1.1)		
Does not know	32 (2.9)	3.0 (1.4)			3.8 (1.4)			3.8 (1.4)			3.6 (1.3)		
Any COVID-19 infected workmate													
Yes	742 (68.4)	2.8 (1.3)	5.099 **	0.01	3.7 (1.2)	5.123 **	0.01	3.6 (1.1)	2.619	0.01	3.4 (1.1)	5.186 **	0.01
No	280 (25.8)	3.1 (1.3)			4.0 (1.3)			3.8 (1.2)			3.6 (1.1)		
Does not know	63 (5.8)	2.8 (1.2)			3.8 (1.3)			3.7 (1.1)			3.4 (1.1)		

* *p* < 0.05; ** *p* < 0.001; M = mean; SD = Standard Deviation; S = statistical; ES = Effect size.

## Data Availability

All data is available within this article.
